# Association between Drinking Habits and Oral Symptoms: A Cross-Sectional Study Based on Japanese National Statistical Data

**DOI:** 10.1155/2020/8874587

**Published:** 2020-12-08

**Authors:** Takeshi Kamoda, Akira Komatsuzaki, Sachie Ono, Satoshi Tanaka, Yasuno Yokoi

**Affiliations:** ^1^Department of Preventive and Community Dentistry, The Nippon Dental University, School of Life Dentistry at Niigata, Niigata City 15103/951-8151, Japan; ^2^Department of Pediatric Dentistry, The Nippon Dental University, School of Life Dentistry at Niigata, Niigata City 15103/951-8151, Japan; ^3^Oral Environment and Community Dental Health, The Nippon Dental University Graduate School of Life Dentistry at Niigata, Niigata City 15103/951-8151, Japan

## Abstract

**Aim:**

The purpose of this study was to investigate the association between drinking habits and subjective symptoms of the oral cavity based on Japanese national statistical data.

**Methods:**

The subjects were 8,698 respondents of the Japan National Livelihood Survey 2013, in their 30s to 60s. The association between drinking habits and each survey item was investigated by contingency table analysis and binary logistic regression analysis.

**Results:**

The proportion of people with drinking habits was 55.3% among men and 20.3% among women, and the proportion of men with drinking habits above the age of 40 years was high. Contingency table analysis indicated an association between drinking habits and the following items in men: subjective symptoms (*p* < 0.01), chewing difficulty (*p* < 0.05), subjective impression of health (*p* < 0.01), smoking habit (*p* < 0.01), and income level (*p* < 0.01). Analysis indicated an association between drinking habits and hospital visits (*p* < 0.01) and smoking habits (*p* < 0.01) in women. When comparing the response rates of symptoms and presence of disease stratified according to drinking habits, inflammatory and sensory system symptoms were common in those who had drinking habits, as were diabetes and gout. Binomial logistic regression analysis with drinking habit as the objective variable indicated statistically significant odds ratios for smoking habit (2.13; 95% CI: 1.65–2.75), difficulty in chewing (1.63; 95% CI: 1.01–2.62), and working hours (1.50; 95% CI: 1.10–2.04). This study identified a correlation between drinking habits and masticatory disorders, suggesting that the effects of drinking as a risk factor for dental diseases should be examined in greater detail in the future.

## 1. Introduction

The purpose of this study was to investigate the association between drinking habits and subjective symptoms related to the oral cavity based on Japanese national statistical data. It is internationally recognized that habitual consumption of alcoholic beverages increases the risk of developing heart disease, cerebrovascular disease, malignant neoplasms, and liver disease [1]. In recent years, the relationship between obesity and drinking, which is a background factor for many lifestyle diseases, has been clarified [2], and even in Japan, the government is calling for appropriate drinking behavior through health measures [3]. However, even moderate drinking has negative social and psychosocial effects. Additionally, alcohol intake varies by country depending on food habits [4].

In Japan, although there have been many studies on the effects of alcohol consumption [5], few studies have evaluated the effect of alcohol on diseases of the oral cavity [6]. A question about drinking was included for the first time in the latest national livelihood survey, which is an approved data source. We obtained anonymous data files from the survey database, with the permission of ministries.

In this study, in order to comprehensively examine the effects of drinking habits on symptoms related to the oral cavity, an exploratory analysis was conducted using the Japanese national statistical database.

## 2. Materials and Methods

### 2.1. Study Population

An anonymized datasheet of the 2013 National Livelihood Survey Questionnaire (Household Survey and Health Survey) was obtained from the Ministry of Health, Labour and Welfare through routine procedures. 8698 survey respondents (4,208 males and 4,490 females) aged 30 to 60 years were included in the current study. The reason for limiting the subjects to those over 30 years old is this period being when the loss holder rate increases according to a dental disease fact-finding survey [7] conducted at the same time. In addition, this age corresponds to the period when the number of people with lifestyle-related diseases increases significantly.

### 2.2. Study Design

In order to take advantage of the large scale of the data of national surveys and to explore the characteristics of factors related to drinking habits, this study was analyzed in two stages, as shown in Table 1.

As the first stage, contingency table analysis was conducted according to sex groups with and without drinking habits. Drinking habits were defined based on the frequency of drinking, using a cutoff frequency of 3-4 times or more per week, and the amount of drinking. Regardless of the frequency of drinking, those who consumed more than the high-risk amount of liquor (equivalent to 40 g or more of pure alcohol per day) specified in the Japanese National Nutrition Survey [7] were considered to have a drinking habit. Also, based on a comparison of the incidence of each symptom and disease condition listed in the survey between groups with and without a drinking habit, differences in trends were confirmed.

The items evaluated in the analysis were history of illness (disease name from ICD-10), lifestyle consciousness, subjective symptoms (bone, muscle, sensory organs, endocrine, mental, and whole body symptoms), awareness of worries/stress, and working hours, among other items, as shown in Table 1.

As the second stage, binomial logistic regression analysis was performed by the stepwise method, with drinking habit as the objective variable, other variables as the explanatory variables, and those with valid responses to all analysis items as the analysis targets (*n* = 1740). A path model was constructed to explore the relationships between the final model variables.

### 2.3. Statistical Analysis

Microsoft Excel 2010 (Microsoft; Tokyo, Japan) and Excel Statistics 2012 (Social Information Services, Tokyo) were used for the aggregate analysis. To test for statistical significance, the *χ*^2^ test was used for contingency table analysis, the Friedman test was used for average rank difference tests, and the partial correlation coefficient significance test was used for binomial logistic regression analysis. In addition, path analysis was performed using the variables used in the logistic regression analysis. The level of significance for judging the difference was set at *p* < 0.05.

### 2.4. Ethical Considerations

The data included in this study were obtained from the database of a national survey conducted in accordance with the survey ethical rules of the Ministry of Health, Labour and Welfare, and the anonymization process was also conducted by the Ministry of Health, Labour and Welfare. This study was conducted with the permission of ministries in accordance with the provisions of Article 36 of the Japanese Statistics Act.

This research was conducted with the approval of the Research Ethics Committee of the Nippon Dental University, School of Life Dentistry at Niigata (license no. ECNG-R-398).

## 3. Results and Discussion

### 3.1. Percentage of People with Drinking Habits

Table 2 shows the gender-related drinking trends among the study population. The analysis showed that 55.3% of men and 20.3% of women were heavy drinkers, and in particular, many men over the age of 40 years had a drinking habit.

### 3.2. Comparison between Drinking Habits and Other Survey Items

Table 3 shows the results of contingency table analysis of drinking habits in relation to other survey items by gender. Items that were found to be associated with drinking habits were the presence or absence of subjective symptoms in men (*p* < 0.01), dental symptoms (*p* < 0.05), subjective health (*p* < 0.01), smoking habits (*p* < 0.01), and household income (*p* < 0.01). In women, presence or absence of hospital visits (*p* < 0.01) and smoking habits (*p* < 0.01) were related to drinking habits.

### 3.3. Symptoms and Diseases Common to People with Drinking Habits

We examined whether there were differences in symptoms and diseases according to the presence or absence of drinking habits, based on their response rates (see Tables 4 and 5). Inflammation and sensory system symptoms were common in those who had drinking habits, as were diabetes and gout.

Comparison of the average rank difference in response rates of each symptom and disease showed a significant difference (*p* < 0.01) in the frequency of both symptoms and diseases in heavy drinkers versus occasional/nondrinkers ranking trends.

### 3.4. Results of Logistic Regression Analysis and Path Model Analysis

Binomial logistic regression analysis with drinking habit as the objective variable showed that smoking habits (odds ratio = 2.13; 95% CI: 1.65–2.75), difficulty in mastication (1.63; 95% CI: 1.01–2.62), and weekly work hours (1.50; 95% CI: 1.10–2.04) were significantly higher than the adjustment variable (gender) (see Table 6).

**Figure 1:**
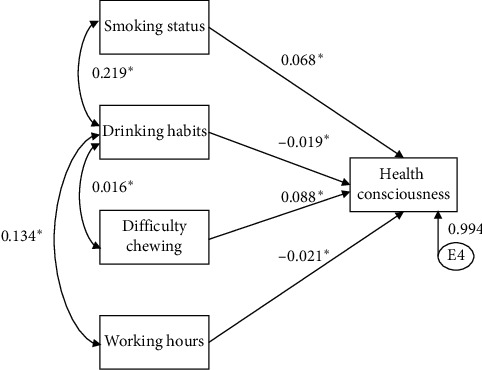
Path model of relationships between variables.

The model that only included significant path coefficients is shown in Figure 1. The direct impact effect coefficients for health consciousness were 0.068, −0.019, 0.088, and −0.021, respectively. The accuracy of the path model was as high as GFI (0.99) and RMSEA (0.06).

## 4. Discussion

It has been pointed out that Japanese drinking habits tend to be different from those in Europe and the US [8], making a simple comparison difficult due to the different types and patterns of alcohol consumption, although the impact of and the motive behind the drinking behavior [9] are considered to be similar to those in other countries.

Cooper et al. [10] proposed a measure of drinking motivation (DMQ-R) based on the results of a review of the literature and reported that drinking motive can be evaluated in terms of three factors: enhance preference, social, and coping.

In this study, there were many people who had a feeling of well-being as well as people who had an adequate income or higher and still had a drinking habit. This suggests that most people consume alcohol for social purposes or as a mood elevator.

On the other hand, there are some items that are associated with drinking behavior for negative reasons. There are similarities between smoking and “difficult to bite” symptoms, as they explain compensatory reasons. Copeland and Brandon [11] reported that drinking and smoking habits (nicotine dependence) correlate with each other for physiological dependence, which supports the results of this study. In terms of oral symptoms, it is believed that alcohol is required to be used for sensory deception such as taste. Masticatory disorders may be similarly affected by alcohol.

Many previous studies on drinking habits pointed out the relationship between stress and drinking [12–16] although the results of the correlation between drinking and stress in our study were unclear. This could possibly be because there are few people who drink alcohol to cope with stress in Japan. Among people we have many social drinking opportunities, it has become more common to drink nonalcoholic beer although there is also a tendency to drink alcohol at events where that is not the purpose. From the results of the National Nutrition Survey [7], it is clear that alcohol consumption by Japanese people has been decreasing over the years. However, world alcohol consumption is increasing. Differences in alcohol consumption trends between Japan and the world are recognized. WHO has promoted “WHO Global Action for the Prevention and Control of NCDs 2013–2020” with the goal of reducing the health damage caused by alcohol by 10% [17].

According to the WHO Global status report on alcohol and health, although there are regional differences, there is a trend towards increased alcohol consumption, which will lead to an increase in the incidence of health issues, such as liver dysfunction [18]. Manthey et al. [1] also warned of an increase in the number of drinkers, stating that the percentage of drinkers is expected to increase by 50% by 2030, and the proportion of temporary high-volume drinkers is also expected to increase by 23%.

Regarding the relationship between dental issues and drinking, according to the WHO's 2007 evaluation [19], alcohol consumption is considered to be a cause of oral and pharyngeal cancers, and drinking habits are considered to be an important instruction target in cancer prevention measures in Japan [20].

Sankaranarayanan et al. [21] pointed out the relationship between alcohol consumption and periodontal disease, and Genco [22] predicted the danger of alcohol drinking habits. There are reports that also highlight the risks of drinking alcohol in terms of dental diseases. However, there are only a few reports on drinking. Therefore, it is assumed that there is an overwhelmingly low chance of giving guidance on alcohol consumption compared to the number of smoking instructions in dentistry. There are many studies on smoking and established guidance methods.

Given the lack of evidence about the relationship between drinking and oral disease, the fact that the relationship between alcohol consumption and masticatory disorders was confirmed in this study is considered to be a remarkable achievement. Masticatory function is affected by blood flow and muscle activity. This basic inadequate evidence was insufficient.

On the other hand, there is a growing social issue related to increasing alcohol consumption by young women in Japan [23]. It has been pointed out that women are more likely to have higher blood alcohol levels than men [24], and there is concern that this might increase the risk of female-specific diseases, such as fetal alcohol syndrome [25].

This study has certain limitations, such as that the data were obtained from a cross-sectional survey, and hence, some points cannot be evaluated universally such as lifestyle habits assessment items. Thus, certain countries' alcohol consumption cannot be compared. In addition, due to the restriction in the survey items in the National Livelihood Survey, the number of survey items related to dentistry was limited.

In future, from the viewpoint of promoting the utilization of national statistical data, it is necessary to review the survey items and promote the integration of multiple national statistical databases.

## 5. Conclusion

From the results of this study, items such as chewing disorders and smoking were clarified as factors related to drinking habits. Our study identified different trends in the response rates of symptoms and diseases in subjects with and without drinking habits.

Binomial logistic regression analysis with drinking habit as the objective variable indicated that the odds ratios were significant for smoking habit, masticatory disorders, and working hours, confirming the relationship between these items and drinking habit.

The observed correlation between drinking and chewing disorders suggests that this relationship needs to be examined in greater detail in future, including the basic lifestyle background mechanism.

## Figures and Tables

**Table 1:** Survey items for analysis.

*Contingency table analysis*	Items

Characteristics	Sex and age group (middle-aged: 30–59 years; older: 60–69 years)
Items related to symptoms	
Subjective symptoms	Presence of subjective symptoms
Dental symptoms	Dental symptoms (tooth pain, swollen or bleeding gums, and difficulty chewing)
Other symptoms	38 general symptoms other than dental symptoms, such as respiratory symptoms
Items related to hospital visits	
Outpatient visits	Current visits
Outpatient visits for dental diseases	Current visits
Disease name	Name of general disease other than dental disease
Items concerning health	
Health consciousness	Subjective health (5 levels, from very good to poor)
Smoking status	Smoking status
Alcohol consumption habits	Heavy drinker and occasional/nondrinker
Lifestyle-related questions	
Economic strength	Poverty level
Income	Total income of household per year
Working hours	No. of working hours per week
Stress-related items	
Awareness of stress	Worries or stress

*Logistic regression analysis*	
Dependent variables	Alcohol consumption habits (heavy drinker: 1; occasional/nondrinker: 0)
Moderator variables	All variables (for each, bad: 1; good: 0)
	Using the stepwise selection method

**Table 2:** Drinking trends of the study group according to age.

		Middle-aged: 30–59 years						Older: 60–69 years			
	Alcohol	30s	%	40s	%	50s	%	60s	%	Total	%
Male	Heavy drinker	437	47.4	536	52.8	614	62.4	742	57.7	2329	55.3
	Occasional/nondrinker	485	52.6	479	47.2	370	37.6	545	42.3	1879	44.7
	Total	922	100.0	1015	100.0	984	100.0	1287	100.0	4208	100.0

Female	Heavy drinker	184	18.6	265	24.5	240	22.4	222	16.5	911	20.3
	Occasional/nondrinker	806	81.4	815	75.5	833	77.6	1125	83.5	3579	79.7
	Total	990	100.0	1080	100.0	1073	100.0	1347	100.0	4490	100.0

Total	Heavy drinker	922	48.2	1015	48.4	984	47.8	1287	48.9	4208	48.4
	Occasional/nondrinker	990	51.8	1080	51.6	1073	52.2	1347	51.1	4490	51.6
	Total	1912	100.0	2095	100.0	2057	100.0	2634	100.0	8698	100.0

**Table 3:** Questionnaire responses stratified by drinking habits.

	Heavy drinker	%	Occasional/nondrinker	(%)	Total	%	*χ* ^2^ test
*Male*							
Subjective symptoms							
Yes	614	52.8	549	47.2	1163	100.0	*∗*
No	1705	56.4	1320	43.6	3025	100.0	
Tooth pain							
Yes	46	55.4	37	44.6	83	100.0	
No	568	52.6	512	47.4	1080	100.0	
Swollen or bleeding gums							
Yes	47	59.5	32	40.5	79	100.0	
No	567	52.3	517	47.7	1084	100.0	
Difficulty chewing							
Yes	34	68.0	16	32.0	50	100.0	^*∗*^
No	580	52.1	533	47.9	1113	100.0	
Outpatient visits							
Yes	911	55.2	738	44.8	1649	100.0	
No	1406	55.5	1129	44.5	2535	100.0	
Outpatient visits for dental diseases							
Yes	110	60.1	73	39.9	183	100.0	
No	801	54.6	665	45.4	1466	100.0	
Opinion about health							
Poor	238	48.4	254	51.6	492	100.0	^*∗∗*^
Regular/good	2083	56.4	1613	43.6	3696	100.0	
Smoking status							
Yes	1000	61.9	615	38.1	1615	100.0	^*∗∗*^
No	1103	49.7	1118	50.3	2221	100.0	
Worries or stress							
Yes	1081	56.5	832	43.5	1913	100.0	
No	1242	54.5	1038	45.5	2280	100.0	
Working hours							
More than 50 hours/week	742	57.3	553	42.7	1295	100.0	
Less than 50 hours/week	1156	56.8	881	43.2	2037	100.0	
Impression of lifestyle							
Harsh	1443	55.0	1179	45.0	2622	100.0	
Regular/comfortable	886	55.9	700	44.1	1586	100.0	
Annual income							
Less than 6 million yen	1135	52.3	1035	47.7	2170	100.0	^*∗∗*^
More than 6 million yen	1194	58.6	844	41.4	2038	100.0	

*Female*							
Subjective symptoms							
Yes	292	18.8	1265	81.2	1557	100.0	
No	617	21.1	2301	78.9	2918	100.0	
Tooth pain							
Yes	12	16.2	62	83.8	74	100.0	
No	280	18.9	1203	81.1	1483	100.0	
Swollen or bleeding gums							
Yes	15	15.3	83	84.7	98	100.0	
No	277	19.0	1182	81.0	1459	100.0	
Difficulty chewing							
Yes	11	16.4	56	83.6	67	100.0	
No	281	18.9	1209	81.1	1490	100.0	
Outpatient visits							
Yes	310	17.3	1480	82.7	1790	100.0	^*∗∗*^
No	600	22.4	2079	77.6	2679	100.0	
Outpatient visits for dental diseases							
Yes	40	14.9	228	85.1	268	100.0	
No	270	17.7	1252	82.3	1522	100.0	
Opinion about health							
Poor	102	19.1	431	80.9	533	100.0	
Regular/good	805	20.5	3127	79.5	3932	100.0	
Smoking status							
Yes	212	38.0	346	62.0	558	100.0	^*∗∗*^
No	657	17.3	3133	82.7	3790	100.0	
Worries or stress							
Yes	490	20.0	1958	80.0	2448	100.0	
No	419	20.7	1605	79.3	2024	100.0	
Working hours							
More than 50 hours/week	76	25.0	228	75.0	304	100.0	
Less than 50 hours/week	536	23.1	1789	76.9	2325	100.0	
Impression of lifestyle							
Harsh	587	21.1	2201	78.9	2788	100.0	
Regular/comfortable	324	19.0	1378	81.0	1702	100.0	
Annual income							
Less than 6 million yen	487	20.1	1933	79.9	2420	100.0	
More than 6 million yen	424	20.5	1646	79.5	2070	100.0	

Note: ^*∗∗*^*p* < 0.01 and ^*∗*^*p* < 0.05.

**Table 4:** Symptoms occurring with the highest frequency according to drinking habits (top ten).

	1st	2nd	3rd	4th	5th	6th	7th	8th	9th	10th

Heavy drinker	Low back pain	Stiff shoulders	Joint pain in hands and feet	Feeling listless	Cough/phlegm	Blocked nose/nasal discharge	Itching	Headache	Blurred vision	Tinnitus
Number (%)	356 (39.3)	310 (34.2)	154 (17.0)	148 (16.3)	131 (14.5)	120 (13.2)	99 (10.9)	97 (10.7)	96 (10.6)	93 (10.3)

	1st	2nd	3rd	4th	5th	6th	7th	8th	9th	10th
Occasional/nondrinker	Stiff shoulders	Low back pain	Feeling listless	Joint pain in hands and feet	Headache	Blocked nose/nasal discharge	Blurred vision	Cough/phlegm	Itching	Numbness of limbs
Number (%)	693 (38.2)	644 (35.5)	339 (18.7)	303 (16.7)	302 (16.6)	251 (13.8)	227 (12.5)	227 (12.5)	221 (12.2)	210 (11.6)

**Table 5:** Frequency of disease that is the reason for hospital visits according to drinking habit (top ten).

		1st	2nd	3rd	4th	5th	6th	7th	8th	9th	10th

Heavy drinker		High blood pressure	Diabetes	Dyslipidemia	Lumbago	Dental disease	Eye disease	Stiff shoulders	Gout	Other skin diseases	Gastroduodenal disease
Number (%)		447 (36.6)	163 (13.3)	158 (12.9)	156 (12.8)	150 (12.3)	101 (8.3)	89 (7.3)	57 (4.7)	56 (4.6)	46 (3.8)

		1st	2nd	3rd	4th	5th	6th	7th	8th	9th	10th
Occasional/nondrinker		High blood pressure	Dyslipidemia	Dental disease	Diabetes	Eye disease	Lumbago	Stiff shoulders	Depression	Allergic rhinitis	Joint disease
Number (%)	558 (25.2)	339 (15.3)	301 (13.6)	291 (13.1)	227 (10.2)	218 (9.8)	175 (7.9)	132 (6.0)	124 (5.6)	114 (5.1)

**Table 6:** Results of logistic regression analysis in relation to drinking habit.

	Partial regression coefficient significance test					95% CI	
Selected explanatory variable	Partial regression coefficient	Wald statistic	*p* value	Judgement	Odds ratio	Lower limit	Upper limit
Sex (male/ref: female)	1.462	152.87	<0.001	^*∗∗*^	4.31	3.42	5.44
Smoking status (yes/ref: no)	0.755	33.43	<0.001	^*∗∗*^	2.13	1.65	2.75
Difficulty chewing (yes/ref: no)	0.486	3.96	0.047	^*∗*^	1.63	1.01	2.62
Working hours (more than 50 hours/ref: less than 50 hours)	0.404	6.64	0.010	^*∗∗*^	1.50	1.10	2.04
Worries or stress (yes/ref: no)	−0.193	2.35	0.126		0.82	0.64	1.06
Health impression (poor/ref: regular, good)	−0.204	2.89	0.089		0.82	0.64	1.03

Note: ^*∗∗*^*p* < 0.01 and ^*∗*^*p* < 0.05. Only explanatory variables selected by the forward selection method are shown. *n* = 1740, coefficient of determination *R*^2^ = 0.134, and percentage of correct classifications = 71.0%; ref: reference.

## Data Availability

The data used to support the findings of this study are available from the corresponding author upon request, only possible to the extent permitted.

## References

[1] Manthey J., Shield K. D., Rylett M., Hasan O. S. M., Probst C., Rehm J. (2019). Global alcohol exposure between 1990 and 2017 and forecasts until 2030: a modelling study. *The Lancet*.

[2] Traversy G., Chaput J.-P. (2015). Alcohol consumption and obesity: an update. *Current Obesity Reports*.

[3] Sugiyama K., Tomata Y., Takemi Y. (2016). Awereness and health consciousness regarding the national health plan “Health Japan 21” (2nd edition) among the Japanese population in 2013 and 2014. *Nihon Koshu Eisei Zasshi*.

[4] Cox W. M., Klinger E. (1988). A motivational model of alcohol use. *Journal of Abnormal Psychology*.

[5] Ohtsuki M., Nishimura A., Kato T. (2019). Relationships between body mass index, lifestyle habits, and locomotive syndrome in young-and middle-aged adults: a cross-sectional survey of workers in Japan. *Journal of Occupational Health*.

[6] Kitagawa M., Kurahashi T., Matsukubo T. (2017). Relationship between general health, lifestyle, oral health and periodontal disease in adults: a large cross-sectional study in Japan. *The Bulletin of Tokyo Dental College*.

[7] Ministry of Health, Labour and Welfare (2020). *General for Statistics and Information Policy Ministry of Health, Labour and Welfare. Handbook of Health and Welfare Statistics 2017: Part 2, Health Status of Alcohol Use, Smoking and Exercise by Year*.

[8] Kojima S., Watanabe N., Takashimazu S. (2019). Effects of changes in drinking habits on lifestyle-related diseases. *Health Evaluation and Promotion*.

[9] Cooper M. L. (1994). Motivations for alcohol use among adolescents: development and validation of a four-factor model. *Psychological Assessment*.

[10] Cooper M. L., Kuntsche E., Levitt A. (2015). Motivational models of substance use: a review of theory and research on motives for using alcohol, marijuana, and tobacco. *The Oxford Handbook of Substance Use and Substance Use Disorders*.

[11] Copeland A. L., Brandon T. H. (2000). Testing the causal role of expectancies in smoking motivation and behaviour. *Addictive Behaviors*.

[12] Sillaber I., Henniger M. S. (2004). Stress and alcohol drinking. *Annals of Medicine*.

[13] Adina T. S., Mihaela-Simona S., Lucia C. P. (2020). The influence of socio-demographic factors, lifestyle and psychiatric indicators on adherence to treatment of patients with rheumatoid arthritis: a cross-sectional study. *Medicina (Kaunas)*.

[14] Becker H. C., Lopez M. F., Doremus-Fitzwater T. L. (2011). Effects of stress on alcohol drinking: a review of animal studies. *Psychopharmacology*.

[15] Becker H. C. (2017). Influence of stress associated with chronic alcohol exposure on drinking. *Neuropharmacology*.

[16] Casey A. (1960). The effect of stress on the consumption of alcohol and reserpine. *Quarterly Journal of Studies on Alcohol*.

[17] WHO (2013). *Global Action Plan for the Prevention and Control of NCD’s 2013–2020*.

[18] WHO (2018). *Alcohol Consumption Global Status Report on Alcohol and Health 2018*.

[19] Petersen P. E. (2008). World health organization global policy for improvement of oral health—world health assembly 2007. *International Dental Journal*.

[20] Sasazaki S., Inoue M., Shimazu T. (2018). Evidence based cancer prevention recommendations for Japanese. *Japanese Journal of Clinical Oncology*.

[21] Sankaranarayanan R., Saxlin T., Knuuttila M., Ylöstalo P., Liisa Suominen A. (2020). Alcohol use and the development of periodontal pockets an 11-year follow-up study. *Journal of Periodontology*.

[22] Genco R. J. (1996). Current view of risk factors for periodontal disease. *Journal of Periodontology*.

[23] Haruyama Y., Nakagawa A., Kato K. (2020). Incidence of metabolic syndrome in young Japanese adults in a 6-year cohort study, the Uguisudani preventive health large-scale cohort study (UPHLS). *Journal of Epidemiology*.

[24] Mancinelli R., Guiducci M. S. (2004). Women and alcohol: biological vulnerability. *Annali dell’Istituto Superiore di Sanità*.

[25] Iwama N., Metoki H., Nishigori H. (2019). Association between alcohol consumption during pregnancy and hypertensive disorders of pregnancy in Japan: the Japan environment and children’s study. *Hypertension Research*.

